# Immersive Robot Teleoperation Based on User Gestures in Mixed Reality Space †

**DOI:** 10.3390/s24155073

**Published:** 2024-08-05

**Authors:** Hibiki Esaki, Kosuke Sekiyama

**Affiliations:** Department of Mechatronics Engineering, Graduate School of Science and Technology, Meijo University, 501-1 Shiogamaguchi, Nagoya 468-8502, Japan

**Keywords:** human–robot interaction, mixed reality, cyber-physical systems, mobile manipulator, virtual reality

## Abstract

Recently, research has been conducted on mixed reality (MR), which provides immersive visualization and interaction experiences, and on mapping human motions directly onto a robot in a mixed reality (MR) space to achieve a high level of immersion. However, even though the robot is mapped onto the MR space, their surrounding environment is often not mapped sufficiently; this makes it difficult to comfortably perform tasks that require precise manipulation of the objects that are difficult to see from the human perspective. Therefore, we propose a system that allows users to operate a robot in real space by mapping the task environment around the robot on the MR space and performing operations within the MR space.

## 1. Introduction

Recently, there has been much work on novel Human–Robot Interaction (HRI) systems for cooperative work between humans and robots. These systems aim to help robots not only more accurately interpret human intentions but also facilitate smoother communication. When working in shared spaces, the synergy between humans and robots is expected to improve work efficiency and safety. The role of augmented reality (AR) and virtual reality (VR) as interfaces in Human–Robot Collaboration (HRC) has received significant attention, highlighting their potential to transform modes of interaction. Zhang et al., demonstrate the effectiveness of VR in training robots for complex manipulation tasks through deep imitation learning and show that VR teleoperation enhances data collection and improves robot performance when executing complex tasks [1]. Sereno et al., provide a comprehensive survey of collaboration in AR, highlighting the importance of AR in enhancing human collaboration and interaction through various aspects such as space, time, and role symmetry [2]. Additionally, Mixed Reality (MR), which combines the capabilities of AR and VR, is believed to revolutionize spatial information processing and the integration of holographic entities within real environments. Ens et al., discuss the evolution of groupware in MR and highlight how MR technology enables richer and more effective collaborative experiences by integrating digital and physical elements [3]. Similarly, de Belen et al., provide a systematic review of collaborative MR technologies, classifying the research into application areas and highlighting the importance of annotation techniques, cooperative object manipulation, and user perception studies in enhancing collaborative MR environments [4].

Expectations have been increasing for human–robot interaction using mixed reality (MR) [5]. One such research is the development of a dynamic object grasping system for human–robot cooperation, which leverages MR to seamlessly integrate real and virtual elements, thereby enhancing the interaction and effectiveness of collaborative tasks [6]. MR extends the capabilities of AR’s virtual overlay and VR’s immersive environments by weaving virtual and physical elements into a cohesive experience. This seamless integration facilitates rich visuals and interactive collaboration within MR spaces, promising a higher level of immersion [7,8]. Research is also being conducted to create a highly immersive experience by directly mapping human movements onto a robot in MR space [9,10]. By directly mapping human movements to robot movements in MR space, humans can control robots through physical intuition. However, if the objects are far from the work area or the robot and the human have different viewpoints, it would be difficult to understand the relationship between the placement of objects and the distance in the physical space by simply reflecting the robot’s movements.

To cope with these issues, we have developed an HRI system in which a human manipulates MR objects based on physical intuition in an MR space and a robot recognizes the changes in the position and posture of the MR objects due to the manipulation. By recognizing changes in the MR objects positions and orientations through such interactions, the robot can realize the user’s intentions in the physical world, paving the way for more flexible and natural manipulation.

In this study, we constructed a basic system framework supposing that different operators share an MR space, perform operations within their respective MR spaces, and realize the results in the physical space. The system of this research operates objects and robots via cyber-space, and if all environmental information is mapped to the cyberspace, it would be equivalent to operating in VR space. However, when completing an operation within a VR space, it is necessary to map all the environmental information related to the task, which can be a difficult problem to realize. This study assumes that the user can directly perceive the main parts of task environment and the robot, while allowing some degree of occlusion. Based on the premise, the proposed system provides information in the MR space that enhances the user’s operational experience. This approach allows the system to avoid the problem of having to map all information in the VR space. This is the advantage of the use of MR space over VR.

In our previous paper [11], we integrated object manipulation in MR space and robot movements in the physical space, allowing the users to intuitively control MR objects. The robot detects the changes in the position and orientation of MR objects based on the user’s manipulations and moves the arm according to the user’s movements in the physical space. Additionally, we visualized real objects as MR objects and built a calibration system that integrates maps between the user and the robot using AR markers and affine transformation.

However, our previous system was functionally limited as the calibration of mapping was possible within 2D space and the position alignment in three-dimensional space was challenging. In this paper, we introduce a calibration method that integrates AR markers, 3D point cloud, and affine transformation, enhancing the accuracy of alignment between MR and the physical spaces. MR functionalities are extended to display the robot’s planning path in MR space, allowing the user to designate or adjust the moving path as needed, thereby overcoming the limitations of the robot’s working range. Furthermore, we improved the user interface to enable more natural and seamless interactions. For example, by adding zoom in/out features, users can adjust the task environment according to their preferences, facilitating easier and more intuitive operations. These enhancements improve the overall efficiency and accuracy of the system, resulting in reducing task completion time and improving task success rates.

## 2. Previous Research

Robots are expected to increasingly support human life in crucial sectors including healthcare, manufacturing, space exploration, and agriculture. Many challenges remain, especially in collaboration with humans. One of the primary obstacles is mutual communication between humans and robots, where robots are often incomprehensible to humans actions and intentions; similarly, humans also might have difficulty predicting robots’ capabilities or intentions. Robots are still unable to infer complex human behavior and need to enhance essential skills to effectively collaborate with humans [12]. The central problem lies in the lack of explicit or implicit information exchange between humans and robots (referred to as gulf of execution and gulf of evaluation within the human action cycle). While humans face difficulty communicating high-level goals to robots in a form that robots can understand, robots have difficulty providing effective feedback for humans to estimate the state of the robot system.

The fields of teleoperation and human–robot interaction (HRI) have evolved significantly with advances in technology. Dinh et al., developed a sensorless force feedback joystick control for the remote control of construction machinery, allowing for more intuitive operation [13]. However, traditional interfaces such as joysticks and keyboards that are less intuitive are still used. These tools create a mismatch between the user’s control space and the device’s workspace, complicating remote operations. Similarly, Truong et al., proposed a force-reflecting joystick control in two-way remote control of construction machinery to improve accuracy and efficiency, while suggesting the complexity of advanced feedback mechanisms [14]. Also, Komatsu et al., point out that remote 2D visualization lacks depth perception, limiting operator performance and reducing immersion and telepresence in remote workspaces [15]. Nakanishi et al., developed an intuitive teleoperation system for human-support robots using VR devices, aiming for a more natural user experience by using a joystick to remotely control the robot arm [16]. Meeker et al., also worked with this approach and showed that even novice operators can intuitively manipulate hands using continuous teleoperation subspaces [17]. However, although the users can quickly learn basic operations using a joystick, it takes time to master the complex operations. Additionally, an immersive workspace is not guaranteed if the operator has to contemplate the robot’s movements during operation.

Remote control systems have used traditional interfaces including joysticks, gamepads, keyboards, and mice. A mismatch between the device’s workspace limits and the user’s control space may complicate remote operations. Also, the lack of depth perception due to 2D visualization of remote sites limits operators’ performance and reduces immersion and telepresence in the remote workspace [18]. Furthermore, controllability would be significantly reduced due to rotational deviations of the display coordinates [19]. Visual technology plays an essential role in safety-critical applications [20]. Therefore, research on in human–robot interaction (HRI) using Mixed Reality (MR) has been advancing to immerse users in the work environment. Bejczy et al., employ MR technology as a new interface to improve remote operation of mobile manipulators in environments where the spread of particulates, chemical substances, and radioactive materials must be tightly controlled to prevent contamination during manufacturing processes [21]. This research demonstrates how MR can improve usability and user experience. Triantafyllidis et al., also researched multimodal interfaces for teleoperation, contributing to improved operability [22]. MR technology has great potential for collocated or remote collaboration [23,24].

MR technology aims to provide a new means of information exchange between users and robots, making collaboration between humans and robots more intuitive and easier to understand. It has great potential to provide users with a more immersive experience and bridge the communication gap between humans and robots by fusing real-world and virtual elements. Research in Human–Robot Interaction (HRI) using Mixed Reality (MR) focuses on enhancing human situational awareness in remote operations and freeing users from physical constraints. MR has been applied to robot remote operation systems to improve remote user perception and enable immersive robot teleoperation (IRT). For instance, Nakamura et al., developed a system that uses VR to support teleoperation of a dual-armed robot, improving task efficiency and accuracy by enhancing user perception and control [25]. Whitney et al., introduced “ROS Reality”, a framework that uses consumer VR hardware to improve remote control immersion and control accuracy, resulting in significant improvements in user performance compared to traditional desktop interfaces [26,27]. For example, MR interfaces overcome the asymmetry between master and slave, and the physical mechanisms constrain robots faced by traditional teleoperation systems, providing operators with more freedom in robot operation. However, MR systems inherently lack haptic feedback and physical structure, which can reduce precise control and maneuverability.

In addition to these issues, HRI using MR technology faces many technical challenges. Firstly, ensuring real-time performance is essential. MR environments require immediate feedback to user actions, as delays can degrade the user experience and lead to operational errors. Furthermore, high-precision real-time transfer and accurate reflection of 3D data are required, which requires high-speed data processing and transfer. Also, it is important to design the user interface such that users can operate it intuitively [28,29]. To overcome these challenges, this study extends our previous calibration method to improve alignment accuracy between MR space and physical space by integrating AR markers, point clouds, and affine transformations. We also propose a novel method for path planning for mobile robot navigation using MR space. This makes it possible to directly visualize and modify the desired path. This gives the user flexible and intuitive control over the robot’s navigation. Finally, the user interface has been improved with the addition of zoom-in/zoom-out functions. This allows the user to tailor their task environment to their preferences and create natural and seamless interactions. These improvements reflect a user-friendly MR space environment and enable intuitive control.

## 3. Immersive Robot Teleoperation System Based on MR Object Manipulation (IRT-MRO)

### Integrating MR Space and Robot System for Human–Robot Interaction

Immersive Robot Teleoperation systems based on MR object manipulation (IRT-MRO) improve the ability to interact with the physical environment by mapping real-world objects into MR space and allowing the user to manipulate objects based on physical intuition.

**Figure 1:**
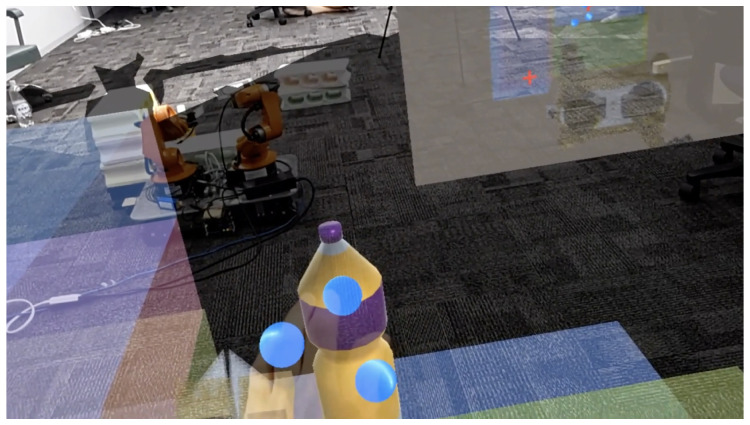
Display image of MR objects during pick-and-place operation.

The system utilizes a Unity-based holographic application with HoloLens2, which is an MR device to reflect real-world objects in MR space, as shown in Figure 1. Users can change the position and orientation of MR objects by directly manipulating them in MR space. By using MR space, it is possible to capture and understand a human behavior action and convert it into spatially meaningful behavior, enabling interaction between physical objects and virtual objects. Interaction in MR space means that when a user performs an operation in MR space, the robot receives the information regarding the human action and takes a physical action, and the user receives the information presented by the robot and performs new actions.

**Figure 2:**
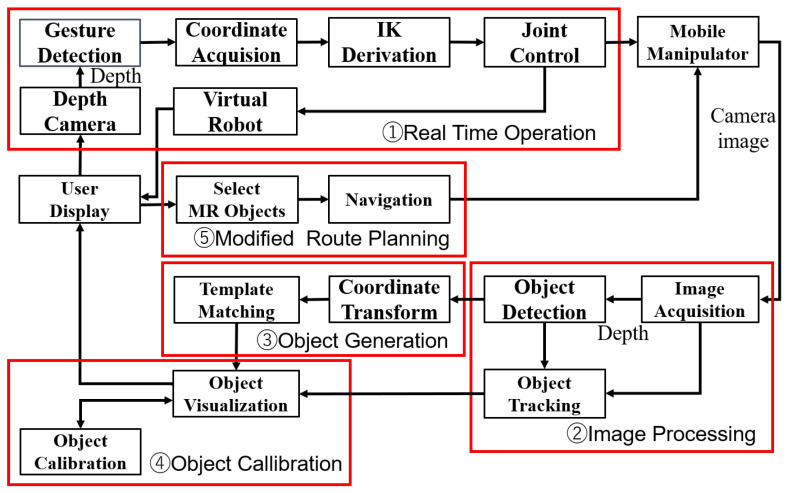
Architecture of the IRT-MRO system composed of three levels of functional space: Physical Space, Cyber-Physical Space, and Cyber Space. Physical Space is the space where users and robots physically exist, including work spaces. Cyber-Physical Space is constructed using MR to abstract the workspace and give informational meaning to the objects. Cyber Space is a complete information space, where user actions and object states are abstracted and exist as information.

The robot employed in this paper is KUKA YouBot, which is a mobile manipulator equipped with a 5-axis robotic arm with a two-finger grip and can move in all directions. On the robot side, changes in the position and posture of objects in MR space are detected and reflected in the robot’s movements in the real space, enabling smoother communication between the human and the robot. In addition, the robot’s movements are reflected on the MR space to provide accurate visual feedback. The system architecture, which consists of four modules, is shown in Figure 2.

**The Real-Time Operation module** tracks the human hand in MR space and obtains the palm position and orientation, converts the coordinates in MR space to the YouBot coordinates, and then calculates the target position and orientation of the YouBot’s end-effector. From the target position and posture, the Levenberg–Marquardt method (LM method) is used to calculate each joint angle in real time.

**The Image Processing module** obtains video from RealSense, an RGB-D camera attached to YouBot’s end-effector. Real-world objects are detected from the acquired video through object recognition. The object detection uses YOLOv5 (You Only Look Once) to obtain the coordinates and size of the detected objects in the camera screen from YOLOv5, and the depth of the object with respect to the center coordinates is obtained using depth information acquired using RealSense, an RGB-D camera. YOLOv5 and DeepSORT are combined to achieve fast and accurate detection and tracking of physical objects. YOLOv5 is suitable for real time processing because it can detect multiple objects simultaneously in a single network, while DeepSORT can track multiple objects across the frames and assign a unique ID to each object. The camera and depth information obtained by the Image Processing module are used to automatically generate MR objects.

**The Object Generation module** converts the camera coordinate system to the ground coordinate system using the coordinates of the object center and depth information detected by YOLOv5. MR objects are then generated with respect to these coordinates prepared in advance for each type of object.

**The Object Calibration module** uses AR markers to correct the scale between MR space and real space and transform the coordinates to the ground coordinate system. Affine transformation is used to improve the coordinate misalignment between real and MR objects.

**The Modified Route Planning module** utilizes MR space to enable users to visualize and modify the robot’s route in real time. Compared to traditional autonomous navigation systems, this module is more adaptable to environmental changes and unexpected obstacles, providing more flexible and intuitive navigation.

In this study, MR space is created using Microsoft HoloLens2 as the MR-HMD. The MR system was developed based on the OpenXR standard, utilizing Unity and MRTK (Mixed Reality Toolkit). Users can perform intuitive illustration actions by manipulating MR objects with hand movements. Additionally, other representations using MR objects can be used to convey the robot’s behavioral intentions to the user.

## 4. Components of Intuitive Robot Operation Based on Mixed Reality Operation (IRO-MRO)

### 4.1. Definition of MR Space by Coordinate Transformation to Real Space

**Figure 3:**
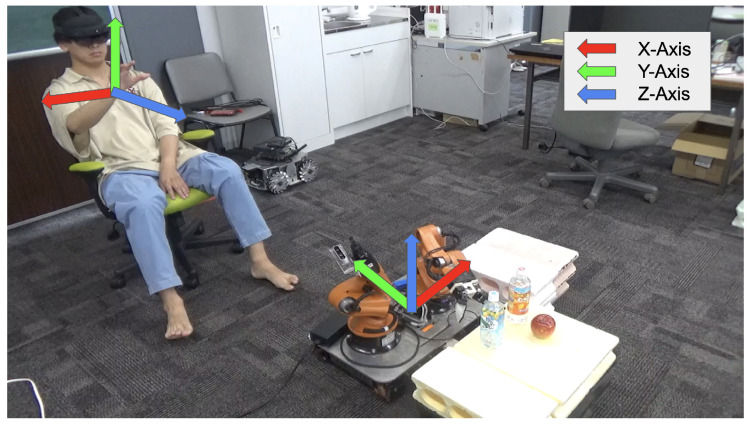
Coordinate systems corresponding to MR space and robot in the physical space.

Figure 3 shows the position and coordinate system of MR space and YouBot. The coordinate system of MR space ${\;\;}^{u}p\in R^{3}$ to that of YouBot ${\;\;}^{r}p\in R^{3}$ is a left-hand coordinate system with the vertical upward direction of y-axis positive in MR space, as shown in Figure 3. YouBot has a right-handed coordinate system defined with the positive z-axis in the vertical upward direction. In the task implementation, for the set of the robots $r=\{r_{1},r_{2},\cdots ,r_{n}\}$ and objects $o=\{o_{1},o_{2},\cdots ,o_{m}\}$, their initial positions are denoted as ${\;\;}^{u}P_{r_{1}}$, ${\;\;}^{u}P_{r_{2}}$, ⋯, ${\;\;}^{u}P_{r_{n}}$ and ${\;\;}^{u}P_{o_{1}},{\;\;}^{u}P_{o_{2}},\cdots ,{\;\;}^{u}P_{o_{m}}$ of robots r and o in the user’s world coordinate system are set for the robot $r=\{r_{1},r_{2},\cdots ,r_{n}\}$ and objects (bottles) $o=\{o_{1},o_{2},\cdots ,o_{m}\}$ in the execution environment. Homogeneous transformation matrix of the robot’s world coordinate system, based on ${\;\;}^{u}P_{r_{n}}$, is calculated as shown in Equation (1). As shown in Figure 3, the coordinate systems of YouBot and MR space are different; so, it is necessary to align the coordinate systems when transferring the information between MR space and robot. The rotation matrix ${\;\;}_{u}^{r}R\in R^{3\times 3}$ of YouBot from the local coordinate system to the world coordinate system is expressed as follows, where $\left(q_{x},q_{y},q_{z},q_{w}\right)$ is the orientation of MR object:

(1) $$\begin{array}{c} {\;\;}_{u}^{r}R=\left[\begin{array}{ccc} 2q_{w}^{2}+2q_{x}^{2}-1 & 2q_{x}q_{y}-2q_{z}q_{w} & 2q_{x}q_{z}+2q_{y}q_{w}\\ 2q_{x}q_{y}+2q_{z}q_{w} & 2q_{w}^{2}+2q_{y}^{2}-1 & 2q_{y}q_{z}-2q_{x}q_{w}\\ 2q_{x}q_{z}-2q_{y}q_{w} & 2q_{y}q_{z}+2q_{x}q_{w} & 2q_{w}^{2}+2q_{z}^{2}-1 \end{array}\right] \end{array}$$

The robot coordinate system can be expressed by Equation (1) and the translation vector ${\;\;}_{u}^{r}t\in R^{3}$ as follows:

(2) $${\;\;}^{r}P={{\;\;}_{u}^{r}R}^{u}\cdot P+{\;\;}_{r}^{u}t$$

The user updates the coordinate transformation sequentially to control the YouBot using the correct position and orientation in the world coordinate system.

### 4.2. Aligning the Origin of MR User’S Viewpoint and Real Robot Using AR Markers

**Figure 4:**
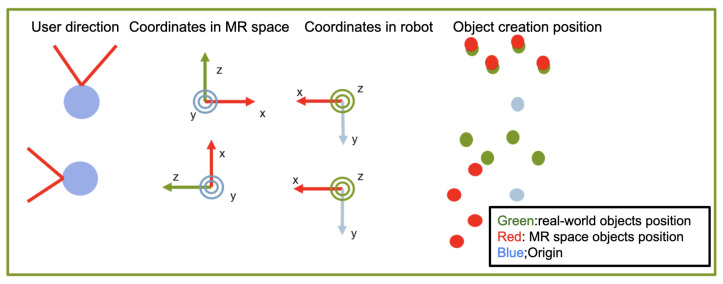
Relation of local coordinate systems between HoloLens2 and YouBot.

In the initial setup, the origin of MR space and the origin of real space must match. With the origin of MR space defined as the origin on the HoloLens2 camera, MR objects are generated in MR space using coordinates from the origin. However, when a user makes an action to grasp an MR object with his/her arm it will cause a misalignment between the MR objects and the real objects. There are two main causes for this misalignment. The first cause is the misalignment of the object when the user sets the origin. If the MR object was placed at the origin of YouBot coordinate in order to match the user and robot coordinate origins, the position of the MR object will change depending on the robot’s movement and the MR object’s position. This tends to cause a misalignment of the position from the origin. The second cause is due to the difference in the timing of setting the origin of HoloLens2 and YouBot. HoloLens2 automatically sets its coordinate origin when the application is launched. Therefore, it was necessary to launch the application by aligning the orientation of the user and the robot. In this way, as shown in Figure 4, the orientation differs between MR space and real space depending on the orientation each time the application is started.

By using AR markers, this discrepancy can be resolved by automatically generating MR objects in MR space based on the AR markers, and having the real robot move based on the AR markers.

**Figure 5:**
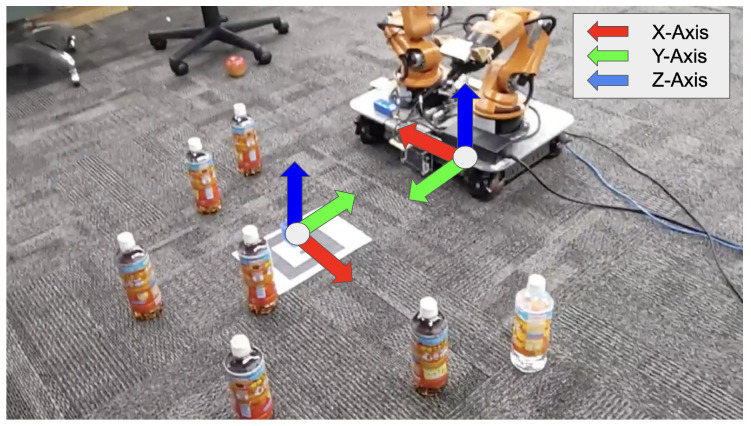
Origin alignment using AR markers.

In this research, the center coordinate of the robot is taken as the origin of the robot, and the position and orientation at which the application is started are taken as the origin of the MR space. The origin of the robot and the origin of the MR space are transformed into a global coordinate system with the floor as the origin. Figure 5 shows the position and coordinate system of YouBot and the position and coordinate system of AR markers. In MR space, Vuforia developed by PTC Inc. is used to detect AR markers. In real space, we use AR_Track_Alvar, an open-source AR tracking framework. To align the MR space with the coordinate system of YouBot, we calculate the rotation matrix and translation vector using the previously presented formulas and perform a coordinate transformation. This ensures that the MR space and YouBot’s position and coordinate system match. The coordinate system of the AR marker is used as the coordinate system of the MR space, and the coordinate system of the real robot is fixed so as to match the coordinate system of the AR marker and the real robot.

When the position of the AR marker is given as $p_{ar}$ and its orientation as $q_{ar}$, and the position of YouBot is denoted as $p_{yb}$ and its orientation as $q_{yb}$, the following transformation formula is applied.

(3) $$\begin{array}{cc} \; & p_{yb}=R_{ar2yb}\left(p_{ar}-p_{ar2yb}\right) \end{array}$$

(4) $$\begin{array}{c} q_{yb}=q_{ar}\cdot q_{ar2yb} \end{array}$$

where $R_{ar2yb}$ is the rotation matrix from the YouBot coordinate system to the AR marker coordinate system and $p_{ar2yb}$ is the transformation of the origin of the YouBot coordinate system to the AR marker coordinate system. In this way, the coordinates are transformed so that the origin of the MR space and the origin of the real space match.

### 4.3. Automatic Generation of MR Objects

In a task environment, there are parts that are easy for the user to directly recognize and parts that are not. The purpose of system is not to pursue the immersive VR, but to enhance intuitiveness by effectively presenting information that is difficult for the user to observe. Mapping physical objects into MR space requires closely aligning the virtual environment with the real world, taking into account physical constraints and the relative positioning of objects in MR space. This fusion makes it possible to reproduce real-world operations and movements in the MR space, thereby improving user operability. Moreover, manipulating MR objects as if they were physical objects in MR space, the user’s sense of interaction and operational intuitiveness are significantly improved.

The process from the detection of physical objects in (2) and (3) of Figure 2 to the mapping of the physical environment in the MR space is given below.

(1) **Object detection:** Detect physical objects using YOLOv5 and obtain their labels and bounding box (x,y,w,h).
(2) **Object tracking:** The object tracking algorithm DeepSORT [30] is used to associate the bounding box of each physical object with its unique ID.
(3) **Depth estimation:** Realsense is used to obtain the depth (z) with respect to the center coordinates of the bounding box of each physical object.
(4) **Camera and world coordinate transformation:** Since the bounding boxes are in camera coordinates, the depth information is used to convert the camera coordinates to world coordinates and obtain the world coordinates (x,y,z) of each bounding box.
(5) **World to robot-based conversion:** The camera coordinates are transformed to the base coordinates of YouBot using the same-order transformation matrix for each joint.
(6) **MR object generation:** MR objects are predefined and generated by the MR objects with the same labels of the physical objects.
(7) **Object placement:** The MR object is generated at an angle of 10 to 20 degrees from the horizontal plane, taking into account the user’s natural head movements during interaction.
(8) **Position update:** The location and ID of each physical object are continuously updated and associated with the generated MR object.
(9) **User interaction:** Updates of the position of the physical objects and the MR objects occur only when the user selects an MR object. Updates stop when the user places the MR object and resume when the user selects the MR object again.
(10) **Top-view object placement visualization:** Visualize the arrangement of MR objects in MR spaces from top perspective.

**Figure 6:**
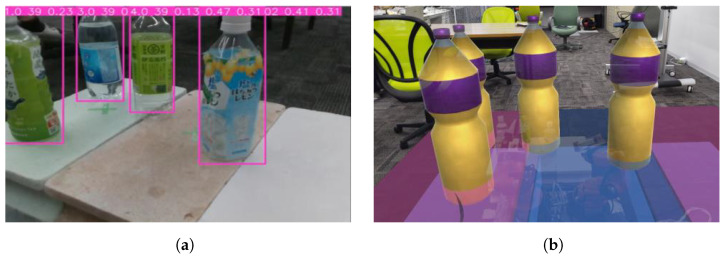
Object Generation in MR Environments Based on Robot Image Recognition: (**a**) Image recognition result obtained from the robot. (**b**) MR object generation based on image recognition (**a**).

This processing flow uses as input the RGB and Depth images obtained from Realsense, attached to the tip of the YouBot’s end-effector, the joint angles of each joint of the YouBot, and the user’s hand movements. Yolov5 is used to detect physical objects (Figure 6a) and DeepSORT is used to track the objects. Using this depth information, the camera coordinates are transformed to world coordinates, the depth center of each bounding box is obtained, and the homogeneous transformation matrix of each joint is used to transform the camera coordinates to YouBot’s base coordinates. In this way, the positional information and IDs of physical objects are placed in the virtual space as MR objects, and the positions and IDs of the physical objects are continuously updated. The update of the position and ID of the physical object stops when the user picks the MR object and resumes when the user selects the MR object again. The MR object is generated at an angle of 10 to 20 degrees from the horizontal plane, taking into account the natural head movements of the user during interaction (Figure 6b). By visualizing the placement of MR objects in MR space, it becomes easier to deal with difficult-to-see placements.

By mapping physical objects into MR space using this method, we bridge the gap between the real and virtual worlds, and clarifying object relationships and physical limitations. This approach not only enhances operational intuitiveness but also enriches the MR experience, making it more immersive and user-friendly.

### 4.4. Map-Integrated Calibration by Manipulating MR Objects in MR Space

#### Point Cloud Synthesis

**Figure 7:**
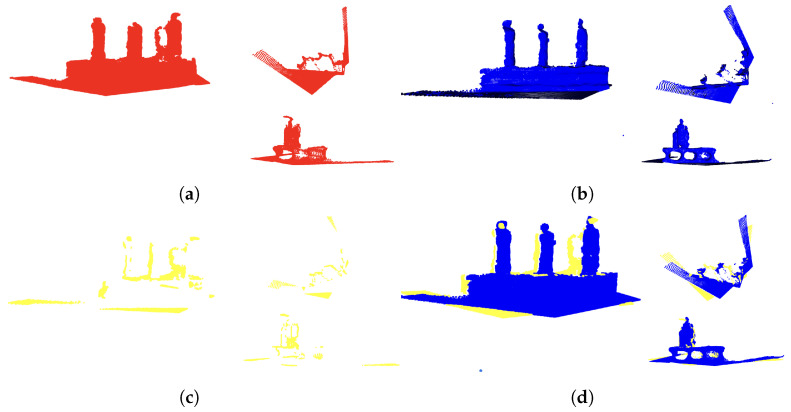
Point cloud bonding flow: (**a**,**b**) are raw point clouds, (**c**) is the non-overlapping point cloud of the two point clouds, and (**a**,**b**,**d**) is the point cloud combining (**a**,**c**).

This section describes the process for synthesizing consecutive point cloud data acquired from Intel RealSense devices. This process is designed to generate a real-time 3D scan of the environment and consists of the following steps:

(1) **Point cloud acquisition and preprocessing:** The initial point cloud Q is acquired at the start of the scanning process from the RealSense device, and each subsequently acquired point cloud is denoted as P. Preprocessing steps such as noise removal are applied to improve data quality, resulting in processed point clouds $P^{\prime }$ and $Q^{\prime }$.
(2) **Global registration:** Global registration is performed between the preprocessed point clouds $P^{\prime }$ and $Q^{\prime }$ using the Random Sample Consensus (RANSAC [31]) algorithm to achieve a coarse alignment. The transformation $T_{\left(p,q\right)}$, consisting of translation *p* and rotation *q*, is estimated by minimizing the error function $E_{g}$ based on the sampled point set:
  (5) $$\begin{array}{cc} E_{g}\left(T\right) & =\sum_{i=1}^{n}||Tp_{i}^{\prime }-q_{closest(i)}^{\prime }{\left|\right|}^{2} \end{array}$$
  (6) $$\begin{array}{cc} T^{*} & =\underset{p,\;q}{arg min}E_{g} \end{array}$$
  where $p_{i}^{\prime }$ is a point in the point cloud $P^{\prime }$ and $q_{closest(i)}^{\prime }$ is the closest point to $p_{i}^{\prime }$ in the point cloud $Q^{\prime }$.
(3) **Registration refinement:** The Iterative Closest Point (ICP [32]) algorithm is applied to further refine the alignment. Based on the transformation $T^{*}$ obtained from RANSAC, the error function $E_{r}$ is minimized to fine-tune the transformation $T_{refined}$, consisting of translation $p_{refined}$ and rotation $q_{refined}$:
  (7) $$\begin{array}{cc} E_{r}\left(T_{refined}\right) & =\sum_{i=1}^{n}\left|\right|T_{refined}Tp_{i}^{\prime }-q_{closest(i)}^{\prime }{\left|\right|}^{2} \end{array}$$
  (8) $$\begin{array}{cc} T_{refined}^{*} & =\underset{p_{refined},\;q_{refined}}{arg min}E_{r} \end{array}$$
(4) **Extraction of non-overlapping point clouds:** Extracting non-overlapping point clouds U is a critical step in the process of synthesizing and updating point clouds from continuous scans, especially when dealing with devices with limited processing power, such as HoloLens2. HoloLens2 is powerful for mixed reality applications but has limited onboard processing resources. As a result, non-overlapping point clouds must be extracted to ensure real-time performance and responsiveness. Using a KD-tree, for each point $p_{i}^{\prime }$ in the point cloud $P^{\prime }$, the nearest neighbor points in point cloud $Q^{\prime }$ are searched within a certain radius ϵ. If no point is found within this radius, $p_{i}^{\prime }$ is added to the non-overlapping point cloud U
  (9) $$U=p_{i}^{\prime }\in P^{\prime },q^{\prime }\in Q^{\prime },||p_{i}^{\prime }-q^{\prime }\left|\right|>\epsilon$$
(5) **Point cloud merging and delivery:** The non-overlapping point cloud U is merged with the existing point cloud $Q^{\prime }$ and updated by M=Q∪U. Acquired point cloud U is then delivered to the MR device, and the point cloud U is added on the MR space. This process efficiently integrates consecutive point cloud data and enables the generation of detailed real-time point clouds. Figure 7 shows an example of merging two point clouds, where the number of points in the point cloud Q is 122,147 and the number of points in the point cloud P is 117,037, and simply adding them together gives 239,184 data points. On the other hand, the number of points in non-overlapping point cloud U is 14,552, and the number of points in point cloud (d) is 131,589. This shows that the point cloud processing of HoloLens2 has become lighter.

### 4.5. Map-Integrated Calibration

**Figure 8:**
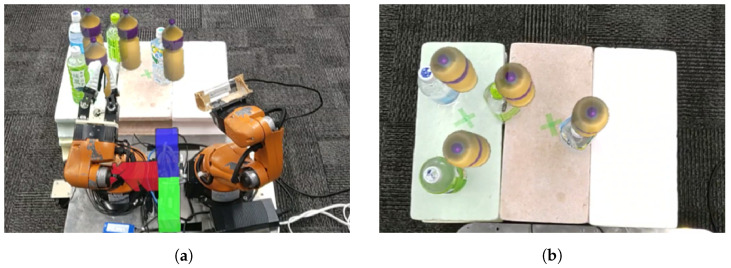
Misalignment of real and MR objects: (**a**) centering a user perspective scene. (**b**) The scene of seen from the top view.

As shown in Figure 8, misalignment of the objects occurs between the real space and the MR space. This can occur for various reasons, such as the difference in depth information, coordinate transformation from the camera coordinate system to the world coordinate system, distortion when the camera is attached, and the difference in scale between MR space and real space. Therefore, if the user performs a task based on physical sensations within the MR space, the robot may not able to properly manipulate the object. To cope with this problem, we propose a calibration system that integrates the MR user’s coordinate system and the real robot’s coordinate system using affine transformation. The origin of the MR space and the robot is based on AR markers.

**Figure 9:**
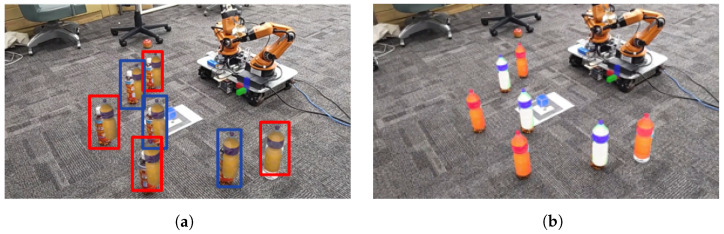
Comparison of the object positions before and after calibration: (**a**) Before calibration. (**b**) After calibration.

As shown in Figure 9a, the affine transformation matrix is calculated using the original coordinates of the object enclosed in a red box and the coordinates of the MR objects when they are superimposed on the corresponding real objects in the point cloud that represents a real-world environment.

**Figure 10:**
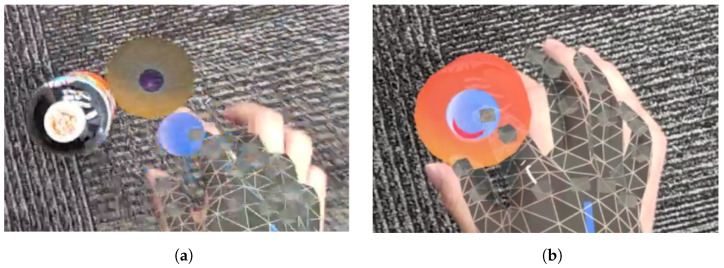
Calibration procedure for object position: (**a**) Before moving MR object. (**b**) After moving MR object.

**Figure 11:**
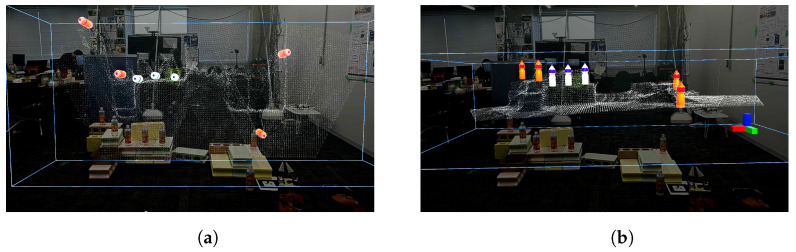
State after position calibration seen in MR space: (**a**) Top view of the state. (**b**) Side view of the state.

**Figure 12:**
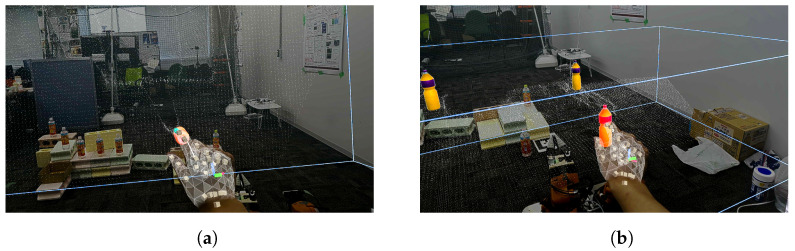
The process of moving MR objects and performing calibration: (**a**) Top view of MR objects being moved for calibration. (**b**) Side view of MR objects under calibration.

When attempting to superimpose MR objects onto real objects, as shown in Figure 10, the user needs to move to the real object’s position, which makes alignment difficult and time-consuming due to visibility issues. Therefore, misalignment may remain uncorrected. By using point clouds and visualizing them in the MR space, the environment can be viewed from various directions, as shown in Figure 11. Overlaying MR objects onto these point clouds allows the user to easily perform calibration, as shown in Figure 12.

Specifically, an affine transformation matrix is generated using the original coordinates $\left(x_{n},y_{n},z_{n}\right)$ of the MR object, obtained through object detection, and the new coordinates $\left(x_{n}^{\prime },y_{n}^{\prime },z_{n}^{\prime }\right)$ when the MR object is detected. MR objects are overlaid onto the corresponding real objects in a point cloud of the real world, as the user performs. This matrix facilitates the calibration of both position and orientation of all objects’ coordinates. The affine transformation matrix for 3D space is given as follows:

(10) $$\left(\begin{array}{c} x^{\prime }\\ y^{\prime }\\ z^{\prime }\\ 1 \end{array}\right)=\left(\begin{array}{cccc} a & b & c & d\\ e & f & g & h\\ i & j & k & l\\ 0 & 0 & 0 & 1 \end{array}\right)\left(\begin{array}{c} x\\ y\\ z\\ 1 \end{array}\right)$$

where a,b,c,e,f,g,i,j,k are the coefficients of scaling and rotation, and d,h,l represent the translation components. Expanding Equation (10) yields

(11) $$\begin{array}{c} x^{\prime }=ax+by+cz+d \end{array}$$

(12) $$\begin{array}{c} y^{\prime }=ex+fy+gz+h \end{array}$$

(13) $$\begin{array}{c} z^{\prime }=ix+jy+kz+l \end{array}\;$$

Substituting the coordinates $\left(x_{n},y_{n},z_{n}\right)$ of the four objects before affine transformation and the coordinates $\left(x_{n}^{\prime },y_{n}^{\prime },z_{n}^{\prime }\right)$ after affine transformation into Equations (11)–(13) gives the coefficients *a* through *l*. To derive these coefficients, a 12-by-1 matrix is given as follows:

(14) $$\left(\begin{array}{c} B_{n}\\ 1 \end{array}\right)=\left(\frac{A_{n}}{0\;0\;0\;\cdots \;1}\right)W$$

where $A_{n}$, $B_{n}$, and W are defined by

(15) $$\begin{array}{cc} A_{n} & =\left(\begin{array}{cccccccccccc} x_{n} & y_{n} & z_{n} & 1 & 0 & 0 & 0 & 0 & 0 & 0 & 0 & 0\\ 0 & 0 & 0 & 0 & x_{n} & y_{n} & z_{n} & 1 & 0 & 0 & 0 & 0\\ 0 & 0 & 0 & 0 & 0 & 0 & 0 & 0 & x_{n} & y_{n} & z_{n} & 1 \end{array}\right) \end{array}\;$$

(16) $$\begin{array}{cc} B_{n} & ={\left(\begin{array}{ccc} x_{n}^{\prime } & y_{n}^{\prime } & z_{n}^{\prime } \end{array}\right)}^{T} \end{array}$$

(17) $$\begin{array}{cc} W & ={\left(\begin{array}{cccccccccccc} a & b & c & d & e & f & g & h & i & j & k & l \end{array}\right)}^{T} \end{array}$$

Using Equations (15) and (16), the affine transformation matrix W is obtained as follows:

(18) $$W={\left(\begin{array}{c} A_{0}\\ A_{1}\\ A_{2}\\ A_{3} \end{array}\right)}^{-1}{\left(\begin{array}{c} B_{0}\\ B_{1}\\ B_{2}\\ B_{3} \end{array}\right)}^{T}$$

Equation (18) gives the affine transformation matrix for the alignment of coordinates.

During the calibration process, as shown in Figure 12, the user selects four different objects to superimpose the MR objects onto the corresponding objects in a point cloud of real space. For all four objects, the pre-overlay coordinates $\left(x_{n},y_{n},z_{n}\right)$ and the post-overlay coordinates $\left(x_{n}^{\prime },y_{n}^{\prime },z_{n}^{\prime }\right)$ are relayed to the robot via ROS#. These steps facilitate the calculation of the affine transformation matrix using the provided data. To aid user understanding, the objects involved in calculation of the affine transformation matrix are highlighted in red and the objects during calibration are marked in blue, as shown in Figure 11. The coordinates $\left(x_{n},y_{n},z_{n}\right)$ of the calibration object serve as the transformation target, and the amount of data can also be adjusted as needed. The coordinates for all objects are transmitted to the robot using ROS#. Using the derived affine transformation matrix, the new coordinates $\left(x_{n}^{\prime },y_{n}^{\prime },z_{n}^{\prime }\right)$ of each object after transformation are calculated according to Equation (18). These transformed coordinates are then converted to AR marker coordinates using Equations (3) and (4), and the resulting coordinates are dispatched to the MR space via ROS#. The object coordinates are updated with these new data. This procedure facilitates integrated map calibration between the user and the robot by interacting with objects within the MR space, ensuring a consistent calibration experience.

### 4.6. Route Planning and Navigation of Mobile Robot Using MR Space

Although the robot is capable of basic autonomous mobile operations and path planning, the user often wants to instantaneously specify or navigate the robot’s position and orientation during remote control. Therefore, there is a need to incorporate the user’s intention into robot autonomous behavior. This paper proposes a novel approach utilizing MR space, enabling the user to directly visualize and modify route navigation of the robot. Unlike traditional autonomous navigation systems, which require complex sensor configurations and sophisticated algorithms to cope with environmental changes and unexpected obstacles, our approach using MR technology allows the users to adjust the routes directly, providing a more flexible and intuitive control over robot operations.

#### 4.6.1. Route Planning Process

**Figure 13:**
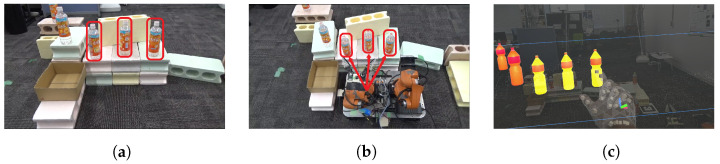
Flow of setting the robot’s target position: (**a**) Target objects for grasping. (**b**) Move to the grasping point. (**c**) Select the MR object to grasp.

**Figure 14:**
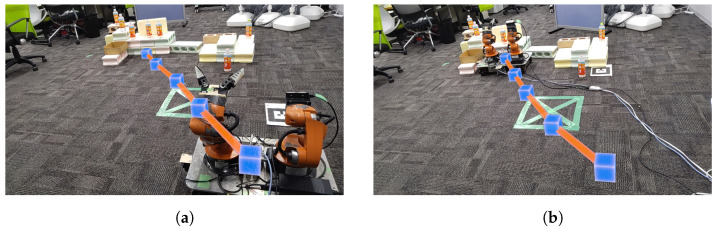
The robot following a route generated in MR space: (**a**) Route generation in MR space. (**b**) The robot following the route.

(1) **Setting the destination:** The user specifies the robot’s goal position using the MR interface. For example, if the user wishes to retrieve three bottles, as shown in Figure 13a, the robot must move to a position (shown in Figure 13b) where it can grasp these bottles. The user selects multiple objects to pick, and the selected objects are highlighted in yellow (Figure 13c). The convex hull is calculated based on the coordinates of the selected objects, and the centroid of this hull is used as the starting point. The optimal position is then calculated, taking into account the maximum reach of the arm and the safety distance to obstacles. The optimal position *p* is represented as follows using the centroid c and search parameters r and θ:
  (19) $$p^{*}=c+r(\cos\theta ,\sin\theta )$$
  where $c=(x_{c},y_{c})$ indicates the coordinates of the mobile base’s centroid, *r* changes within the range of (S≤r≤D − S), and θ ranges from 0 to 2π, defining the direction of the searched position. The optimal position $p^{*}$ is chosen such that the distance from the initial position $p_{0}$ is minimized. This is formulated as the following optimization problem:
  (20) $$\min_{r,\theta }\left|\right|p^{*}-p_{0}\left|\right|$$
  where, $p^{*}$ must be within the maximum reach distance *D* and at least the safety distance *S* away from each object. Also, let P denote the set of coordinates of the selected MR objects, and each coordinate $\left(x_{i},y_{i}\right)$ in *P* corresponds to the position of an individual object selected by the user.
  (21) $$S\leq \left|\right|p^{*}-\left(x_{i},y_{i}\right)\left|\right|\leq D\;\;\;\left(\forall \left(x_{i},y_{i}\right)\in P\right)$$
  By solving this optimization problem, the robot will be able to efficiently and safely access the target object group from an optimal position. This position is the closest to the selected objects, and if multiple optimal positions exist, the one with the shortest distance from the initial position is chosen. This approach allows the users to easily specify the robot’s target location, and the robot automatically calculates the optimal position to efficiently reach the target objects.

(2) **Route planning:** Once the destination is set, the system employs a graph-based environmental model to determine the optimal route from the robot’s current position to the target destination. The Dijkstra algorithm is applied during this process to select the route that minimizes the cost between nodes.
(3) **Route visualization:** The calculated route is visualized in real time in the MR space, allowing the user to see the route in a 3D space through their MR device, as shown in Figure 14.

#### 4.6.2. User-Driven Route Modification

**Figure 15:**
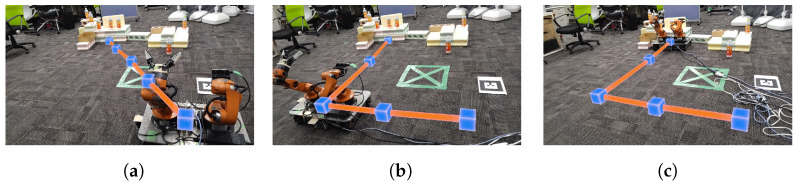
The robot following a route adjusted by the user in MR space: (**a**) Generating a route. (**b**) Adjusting the route. (**c**) Following on the route.

After the route is visualized, the user can interact with the MR space to directly manipulate the waypoints on the route, enabling route modifications, as shown in Figure 15. This process consists of the following steps:

(1) **Initiating route modification:** The user selects specific waypoints they wish to modify using MR device.
(2) **Adjusting way points:** The user directly drags and drops the selected waypoints to new positions in the MR space, modifying the route in real time.
(3) **Updating the route:** When the position of the waypoint is changed, the system automatically recalculates the route and updates the new path and displays in the MR space.

Once the user completes the route modification, the system immediately communicates the new route to the robot; then, the robot efficiently moves towards the target destination following the updated path. In this study, we utilize Lidar SLAM for mapping dynamic environments during robot movement. This process demonstrates the system’s capability to swiftly respond to a sudden environmental change and occluded obstacles from the user. This approach allows the user to experience interactions that cross the boundaries between the real and virtual worlds, enabling more intuitive and flexible route planning. If the robot encounters unknown obstacles during autonomous movement, the user can intervene in real-time and adjust the robot’s path accordingly. This ensures that the robot can be controlled as intended by the user even if the mapping is incomplete.

### 4.7. Real-Time Operation of Robot Arm by Hand Tracking

The HoloLens2 tracks the user’s palm position and orientation, setting them as the target for the robot’s end-effector. A pinching gesture by the user commands the gripper to close. The robot in the MR space is controlled via ROS#, facilitating communication between the Unity-based holographic application on HoloLens2 and the robot’s nodes.

The data sent from HoloLens2 to YouBot include the position and orientation of the back of the hand, which determine the target state of the end-effector, and the air tap gestures to control the gripper’s open and close actions. This positional and orientation data are transformed into coordinates for the end-effector’s target state using the Levenberg–Marquardt method (LM method) [33] to update each joint angle:

(22) $$\theta_{k+1}=\theta_{k}+{\left(J^{T}J+\lambda D_{k}\right)}^{-1}J^{T}e\left(\theta_{k}\right)$$

**Figure 16:**
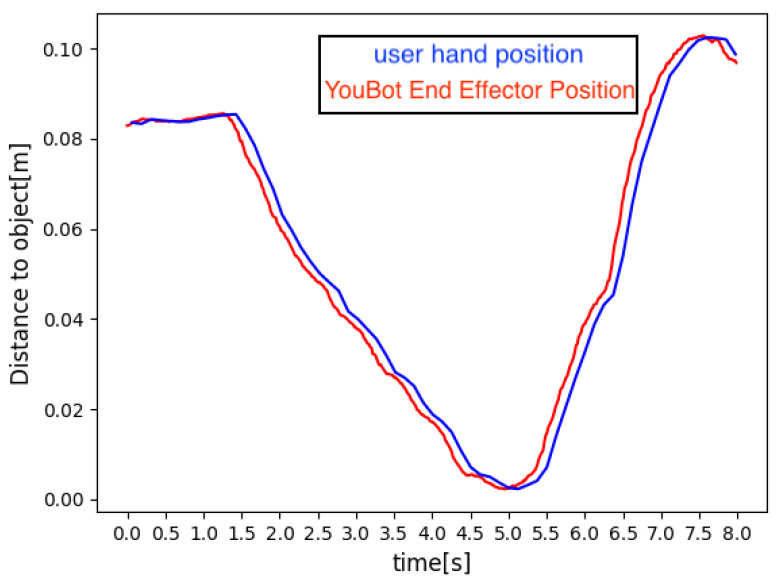
Control response of YouBot arm following user’s hand movement.

In Equation (22), $\theta_{k}$ represents the joint angles’ estimated vector at iteration *k*. J is the Jacobi matrix for the error vector $e(\theta_{k})$, which indicates the discrepancy between the position of the robot arm and the target position. λ is the damping factor, and $D_{k}$ comprises $J^{T}s$ diagonal elements. If YouBot and MR space have different coordinate systems, the transformation using Equation (1) will accurately update YouBot’s joint angles in MR space and provide accurate visual feedback. For gripper operation, the coordinates of the user’s left index finger and thumb tips are monitored. The system sends *open* and *close* commands based on the distance variation between these two points, controlling the gripper’s action. Once a command is received, the gripper’s servo motors operate accordingly. This interactive system enables intuitive robot control through user gestures, assigns spatial significance to these gestures, and enhances the user–robot interface. Additionally, to ensure safety under real-time control, we restrict the movements of the robot arm to avoid collisions with other mechanisms and structures. The system’s real-time response to a user movement was validated, demonstrating that the YouBot arm can track the motion of the user hand within 0.2 s, as illustrated in Figure 16. The red line represents the position of the user’s hand and the blue line represents the position of the end-effector of YouBot’s arm. This rapid response time allows target coordinates and orientations to be seamlessly shared between the user and the YouBot arm in MR space. Consequently, the user can monitor the movements of the YouBot arm in the real world while operating within the MR environment, simplifying the execution of complex tasks. Moreover, the shared spatial context between the user and the robot gives gestures meaningful spatial meaning, making gesture-based control of the robot more intuitive. This symbiosis of user and robotic movements within a shared space highlights the effectiveness of the system in facilitating natural, efficient human–robot interaction.

## 5. Verification of Alignment between MR Objects and Real Objects

### 5.1. Experimental Setup

**Figure 17:**
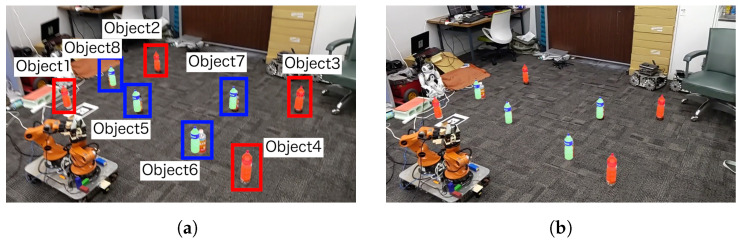
Experimental environment for verifying object position: (**a**) Before calibration. (**b**) After calibration.

Eight objects were placed, and the algorithm above (9) was used to verify the misalignment between the MR object and the real object five times. The objects were all the same type of plastic bottles and placed randomly. The object circled in red in Figure 17a is the object used to calculate the affine transformation matrix. The selection of the objects for calculating the affine transformation matrix is determined by the user. The user operates the system so that the objects surrounded by a red frame are superimposed on the real objects. The four objects surrounded by a blue frame are the targets for position calibration. The number of objects surrounded by the blue frame can be increased or decreased. The coordinates of the objects are based on the coordinates of the AR markers. The AR markers are placed in positions where the robot can detect them without moving.

### 5.2. Experimental Results

**Figure 18:**
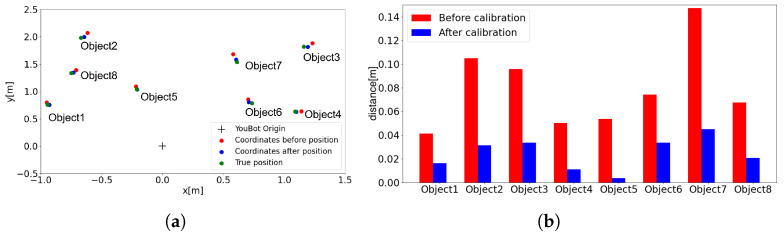
Position calibration results using MR objects: (**a**) Position error of objects. (**b**) Distance error of objects.

Figure 18a shows the results of the verification of the positional misalignment between the MR object and the real object. The origin of YouBot (black), the coordinates before transformation (red), and the coordinates after transformation (blue) are shown. Figure 18b shows the discrepancy between the position of the real object and the MR object. Figure 18b (1–4) present the MR objects manipulated by the user to obtain the affine transformation matrix. Columns 5–8 show the positions of MR objects calibrated using the affine transformation matrix. However, the position of the object after the transformation is closer to the actual object coordinates than before the transformation; therefore, by using AR markers and the affine transformation, it is confirmed that the discrepancy between the MR object and the real object is reduced.

Although this calibration system is very intuitive, there may be some misalignment depending on a person’s line of sight. Calibration of objects on a plane is easy, but it is difficult to superimpose MR objects on real objects in the 3D direction; so, it needs to be improved to simplify the calibration in the 3D direction.

## 6. Verification Using Pick-and-Place Experiments

### 6.1. Experimental Setup

**Figure 19:**
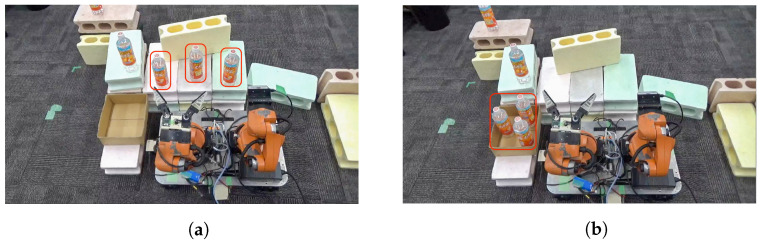
Experimental setup for sequential pick-and-place operation for three bottles into adjacent box: (**a**) Before operation. (**b**) After operation.

**Figure 20:**
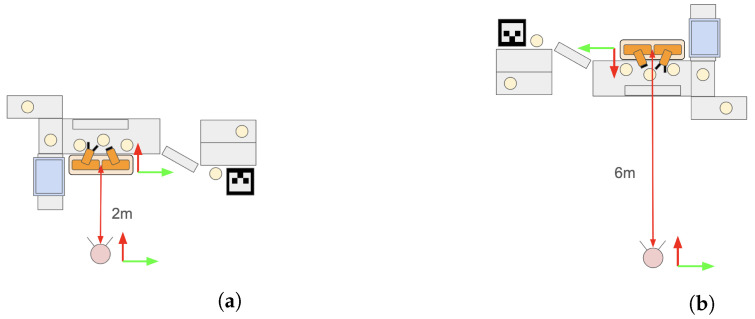
Overview of experimental condition with different placements of user, robot, and objects for operation: (**a**) Arrangement where the user and the robot look in the same direction. (**b**) Arrangement where the user and the robot look in opposite directions.

Figure 19 illustrates an example of a pick-and-place experiment using the proposed system. In this task, the robot is required to place three randomly positioned bottles within its grasp range into an adjacent box. The task is considered successful if all three bottles are correctly placed in the box. This study supposes that the user can perceive the task environment. Additionally, we restrict the task area, and the point cloud data volume is uniformly reduced to avoid affecting the sense of operation. As shown in Figure 20, experiments were conducted to evaluate the effects of different perspectives and the distance between the user and the robot. Specifically, we deal with two experimental conditions where (1) the user and the robot are facing the same direction at a distance of 2 m (Figure 20a) and (2) the user and the robot are facing different directions at a distance of 6 m (Figure 20b). These experiments aim to assess how the differences in perspective and the distance between the user and the robot affect the performance of the pick-and-place tasks.

**Figure 21:**
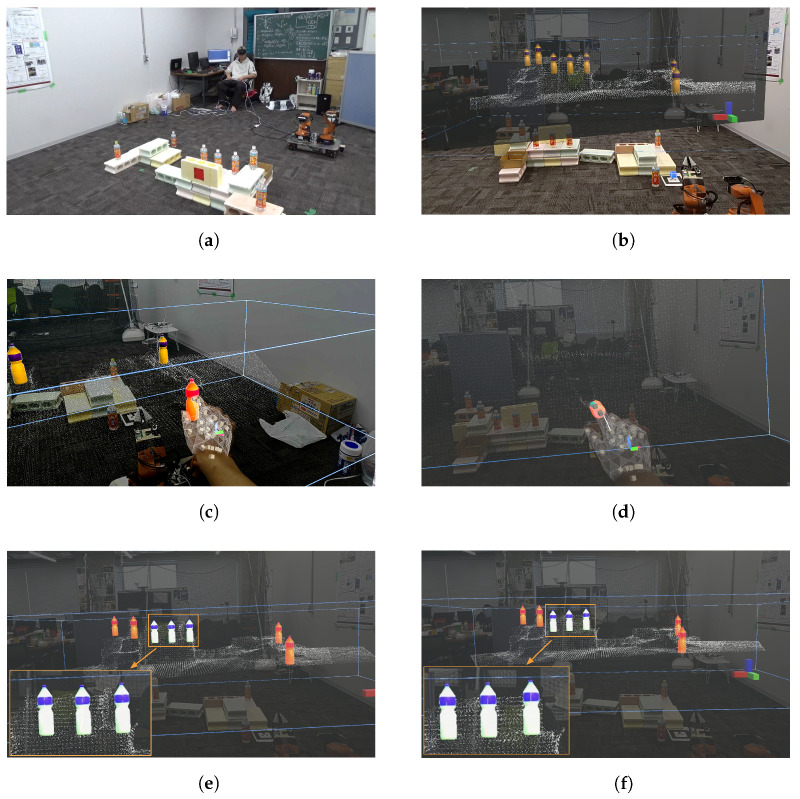
Experimental snapshots of object position calibration from the user perspective: (**a**) Robot acquiring point cloud and MR objects from the third person’s perspective. (**b**) Acquired the point cloud and MR objects from the user’s perspective. (**c**) Position alignments performed in the MR space from a side perspective. (**d**) Position alignments performed in the MR space from a top perspective. (**e**) Before performing affine transformation. (**f**) After performing affine transformation.

**Figure 22:**
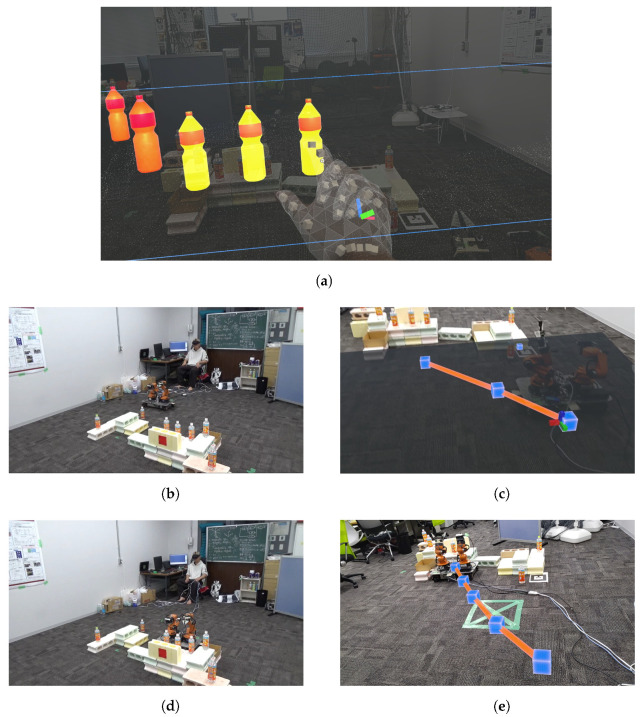
Experimental snapshots of robot route generation and locomotion from the user perspective: (**a**) Touching the MR objects to designate the goal position to the robot. (**b**) Route generation by robot connecting between current position and goal position. (**c**) Visualizing the route corresponding to (**b**) in MR space. (**d**) Moving the robot along the route in real space. (**e**) Robot following the route visualized in MR space.

**Figure 23:**
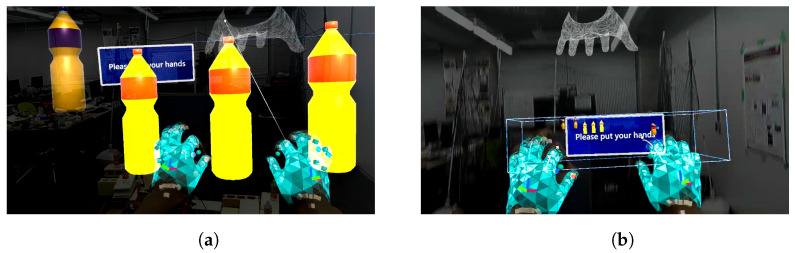
Scaling of MR objects and environment from the user perspective: (**a**) Zooming-in of MR objects by the user. (**b**) Zooming-out of MR objects by the user.

**Figure 24:**
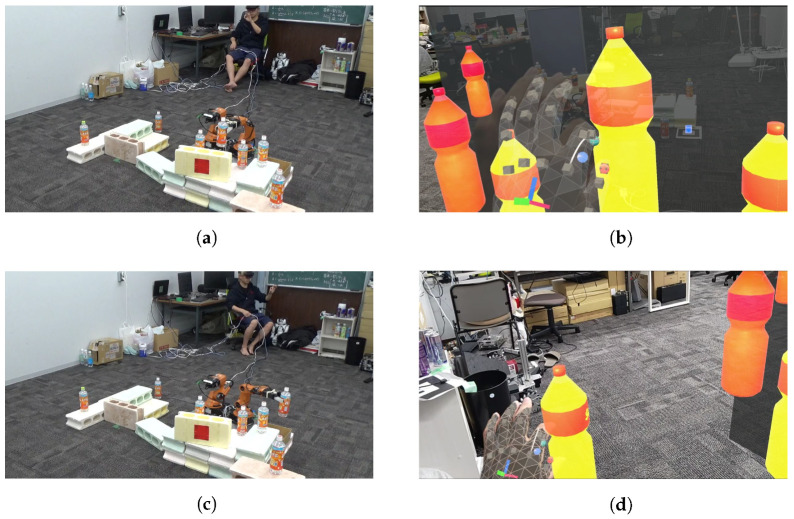
Experimental snapshot of sequential pick-and-place experiment: (**a**) Robot grasping the real object. (**b**) User grasping the MR object. (**c**) Robot placing the real object. (**d**) User releasing the MR object.

As shown in Figure 21a,b, the point cloud and objects are acquired first. Then, the viewpoint is switched between a side view and a top view, as shown in Figure 21c,d, aligning the positions of four objects in the point cloud (real space). The affine transformation matrix is obtained using the positions of four objects before and after the movement, and the positions of the other three objects that have not been moved are corrected. The positions of the objects before calibration are shown in Figure 21e and the positions after calibration are shown in Figure 21f. Next, as shown in Figure 22a, the object to be moved is touched in the MR space. The position information of the touched object is used to calculate the goal position of the robot to move and a path is generated, as shown in Figure 22b,c. The user can adjust the generated path in the MR space. Then, the robot moves along the path to the task environment, as shown in Figure 22d,e. The user can adjust the MR space by scaling and rotating it to achieve a suitable MR space for performing operations, as shown in Figure 23. When the user grasps the MR object, as shown in Figure 24a,b, the robot also grasps the real object. Similarly, when the user releases the MR object, the robot also releases the real object, as shown in Figure 24c,d. This procedure is used to perform the pick-and-place task. In order to verify whether the user can operate the robot intuitively, the task is repeated about 10 times. We evaluate the success rate and the task execution time from the start of the task.

### 6.2. Experimental Results

**Figure 25:**
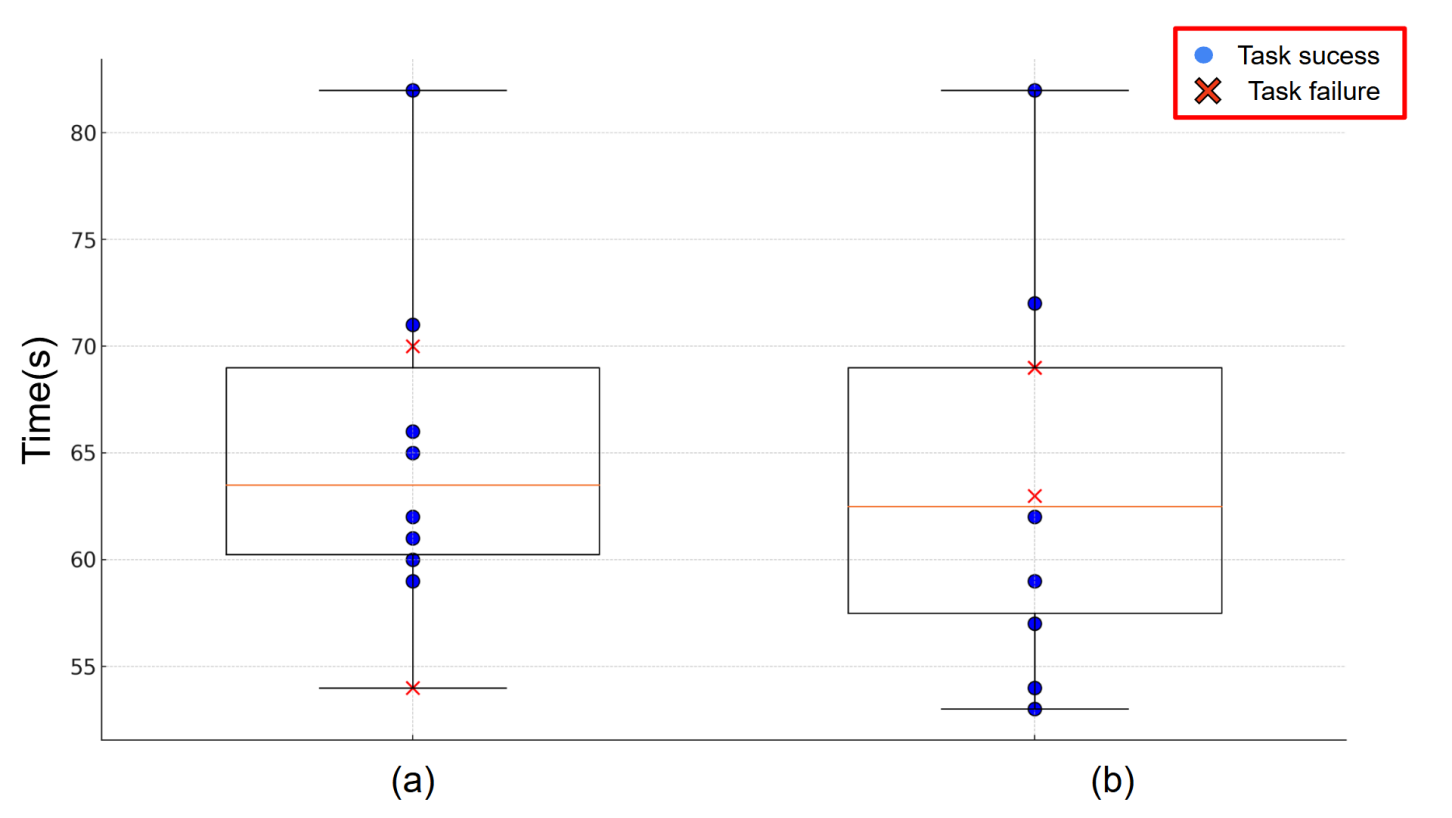
Comparison results of two conditions of pick-and-place in terms of task completion time and success rate: (**a**) The same direction at 2 m distance. (**b**) The different direction at 6 m distance.

Figure 25 shows the box-and-whisker plot of the comparison that visualizes the distribution of task execution time for the pick-and-place experiments conducted under two different conditions. The box-and-whisker plot displays the minimum, first quartile, median, third quartile, and maximum values of the execution time, providing a comprehensive summary of the data distribution. In this figure, the circles indicate the successful trials (task success) while the crosses indicate unsuccessful trials (task failure). As shown in Figure 25, in the case of experiment (a), which was conducted from the same perspective and at a near distance to the robot, the average execution time was 65.0 s and the maximum execution time was 82 s. The success rate of case (a) was 80%. On the other hand, in experiment (b), conducted from a different perspective and at a far distance to the robot, the average execution time was 64.0 s and the maximum execution time was also 82 s. The success rate of case (b) was 70%.

The experimental results in Figure 25a show significant improvements in the operation of the pick-and-place system when calibration and environmental scaling are employed. Also, the results in Figure 25b confirm that changes in viewpoint and distance do not significantly affect task success rate or completion time. From this result, we conclude that the MR system can effectively control the robot from the user’s perspective.

Proposed calibration improved accuracy of mapping the physical environment to the MR space. The experimental results confirm that the tasks can be easily accomplished if the position error is within 5 cm. In terms of operability, we confirm that the control response of the robotic arm was sufficient according to the user’s operating feel. Furthermore, adjusting the scale for the work environment significantly improves robot control, especially in pick-and-place tasks that require meticulous coordination. Consequently, both the success rate and the required task completion time were improved, validating that users can operate the system more intuitively and effectively.

A primary cause of some task failures is due to the use of the LM method, which updates each joint angle of the robot arm based on its current and target positions. In our robot, the trajectory of the arm to avoid collisions between the arm and the surroundings has not yet been considered. Therefore, the robot arm occasionally collides with the work environment, resulting in dropping of the gripped bottle.

To expand the task environment and improve calibration accuracy, more detailed and large-scale point cloud data are required. However, there is a trade-off between the data amount for accuracy and processing speed for user experience. To increase feasibility, this issue can be resolved by varying the density of point cloud information depending on the importance of the task environment. While initial experiments on the task of changing object positions have shown promising results, we will expand the scope to scenarios that require more complex operations to verify the effectiveness of our system.

## 7. Conclusions

In this paper, we proposed IRT-MRO system to improve the interaction between humans and robots in the MR space. The IRT-MRO system is built from multiple subsystems, enabling intuitive manipulation of real-world objects within MR space and allowing these changes to be executed by robots in the physical world. First, we developed a system that visualizes real-world objects as MR objects using YOLOv5. This technology allows the user to recognize and manipulate the real objects within the MR space. Next, we developed a map-integrated calibration system that realizes object manipulation on the MR environment by combining AR markers, point cloud, and affine transformation. This system allows the user to manipulate objects within the MR environment, improving the corresponding physical world actions by the robot. Furthermore, we introduced a system that utilizes MR functions to display the robot’s movement path in MR space and make adjustments as necessary. This allows the robot to quickly respond to changes and unexpected obstacles in the environment. Finally, these technologies have been integrated to the human–robot interactive system as a cyber-physical system. The experimental results demonstrated effective remote control of pick-and-place tasks by the mobile manipulator YouBot via the MR space. The IRT-MRO system enhances flexible viewpoint changes and zooming-in 3D space during operation. These effective visual supports with MR information provides the user with physically intuitive and immersive operation.

Although this research assumes that the task environment is visible to the user, it is possible to extend the system to cope with situations where part or all of the task environment cannot be seen. Additionally, future challenges include expanding the diversity of effective interaction target identification and information presentation functions. Thus, the purpose of this system is to enhance focus on tasks by freely adjusting between emphasizing reality in the task environment and representation in cyberspace. This enables users to move beyond physical constraints and manipulate robots with more natural movements. Predictive modeling technology based on user movements will be introduced and leveraged to enhance the robot’s ability to accurately interpret user movements and reflect them in a manner appropriate for task execution. With this technology, we aim to make the robot’s movements more intuitive and aligned with the user’s intentions.

When operating within a MR space, it is important to indicate the user’s operational intent but it is not always necessary to directly control each action. Given the current advances in robotics standards, it is now possible for robots to recognize the user’s intentions and autonomously control their actions based on their surrounding environment. While this research focuses on building a foundational system, it possesses high scalability to address such issues. In fact, the author’s group has been working on this approach [34]. We have developed a system where robots abstract the user’s exemplary actions in MR space and reconstruct their action plans according to their surrounding environment. However, we are aware that there are many unsolved issues regarding the safety of work using MR in situations where humans and robots physically collaborate and it is necessary to deepen the discussion.

Finally, MR-based technologies are highly dependent on the performance of the MR device. Currently, MR equipment slows down and degrades the user experience when mapping a larger area than the current experimental environment. Also, due to the narrow field of view, if the user operates in a position where their hands are not recognized, the device cannot track the hands, reducing operability and becoming a potential safety hazard. These issues will limit the tasks that can be performed. However, in recent years, new models of MR devices have been developed and efforts are being made to improve the processing speed and field. Therefore, it is expected that the level and scale of feasible tasks will undoubtedly increase in the future.

By integrating these systems, we plan to develop seamless interaction between the MR and physical environments, and significantly enhance collaboration work between humans and robots.

## Data Availability

The data presented in this study are available on request from the corresponding author.
